# Evaluation of Physicochemical Properties of Amphiphilic 1,4-Dihydropyridines and Preparation of Magnetoliposomes

**DOI:** 10.3390/nano11030593

**Published:** 2021-02-27

**Authors:** Oksana Petrichenko, Aiva Plotniece, Karlis Pajuste, Martins Rucins, Pavels Dimitrijevs, Arkadij Sobolev, Einars Sprugis, Andrejs Cēbers

**Affiliations:** 1 Laboratory of Magnetic Soft Materials, Faculty of Physics, Mathematics and Optometry, University of Latvia, 3 Jelgavas str., LV-1004 Riga, Latvia; andrejs.cebers@lu.lv; 2 Latvian Institute of Organic Synthesis, 21 Aizkraukles Str., LV-1006 Riga, Latvia; aiva@osi.lv (A.P.); kpajuste@osi.lv (K.P.); rucins@osi.lv (M.R.); p.dimitrijevs@osi.lv (P.D.); arkady@osi.lv (A.S.); 3 Department of Pharmaceutical Chemistry, Faculty of Pharmacy, Riga Stradiņš University, 21 Dzirciema Str., LV-1007 Riga, Latvia; 4 Laboratory of Chemical Technologies, Institute of Solid State Physics, University of Latvia, 8 Kengaraga Str., LV-1063 Riga, Latvia; esprugis@cfi.lu.lv

**Keywords:** 1,4-dihydropyridine amphiphiles, iron oxide nanoparticles, magnetoliposomes, lipid monolayers, physicochemical properties

## Abstract

This study was focused on the estimation of the targeted modification of 1,4-DHP core with (1) different alkyl chain lengths at 3,5-ester moieties of 1,4-DHP (C_12_, C_14_ and C_16_); (2) N-substituent at position 1 of 1,4-DHP (N-H or N-CH_3_); (3) substituents of pyridinium moieties at positions 2 and 6 of 1,4-DHP (H, 4-CN and 3-Ph); (4) substituent at position 4 of 1,4-DHP (phenyl and napthyl) on physicochemical properties of the entire molecules and on the characteristics of the obtained magnetoliposomes formed by them. It was shown that thermal behavior of the tested 1,4-DHP amphiphiles was related to the alkyl chains length, the elongation of which decreased their transition temperatures. The properties of 1,4-DHP amphiphile monolayers and their polar head areas were determined. The packing parameters of amphiphiles were in the 0.43–0.55 range. It was demonstrated that the structure of 1,4-DHPs affected the physicochemical properties of compounds. “Empty” liposomes and magnetoliposomes were prepared from selected 1,4-DHP amphiphiles. It was shown that the variation of alkyl chains length or the change of substituents at positions 4 of 1,4-DHP did not show a significant influence on properties of liposomes.

## 1. Introduction

Scientists worldwide have made many efforts to expand the invention and development of broad range nanoparticle delivery systems. Liposomes have been extensively studied as promising delivery systems due to their efficiency, biocompatibility and dual character, i.e., the ability to entrap either hydrophobic or hydrophilic drugs, improving their pharmacokinetic and pharmacodynamic properties [1,2,3,4]. Magnetic iron oxide nanoparticles are used for different scientific and technological purposes due to their peculiar properties. The main feature of these particles is to enable movement in a magnetic field. Magnetic nanoparticles (MNPs), due to their biocompatibility and functionality, have been considered to be promising for applications in medicine. Their unique magnetic and electric properties allow for their application in magnetic resonance imaging as contrast agents [5] for treatment in hyperthermia [6,7]. Currently, MNPs are studied for cell labelling and separation [2,8]. Magnetic iron oxide nanoparticles have been used, for example, as a trigger drug release from magnetoliposomes (MLs), through a magneto–nanomechanical approach [9], for magnetically guided cells in tissue engineering [2,10,11] when the MNPs can be coated with a polymer or encapsulated inside liposomes producing MLs. Magnetoliposomes are also used for magnetofection and drug delivery by magnetic targeting [12,13,14]. The investigation of MLs’ morphology and physical properties is an important issue. The chemical structure and shape of cationic compounds determine their self-assembling and DNA complexation properties, and hence the gene delivery activity [15]. 

Synthetic nanoparticle-forming cationic lipid-like compounds have been developed as delivery agents for the transfer of genetic materials, including plasmid DNA (pDNA) molecules into cells [16,17] and for therapy and diagnostic applications [18]. In general, among the synthetic cationic delivery systems, quaternary ammonium surfactants are more toxic than their analogues, with the cationic charge delocalized in a heterocyclic ring [19,20,21]. It is important to evaluate liposome forming lipid properties for the development of new liposome systems [22].

Multiple amphiphilic 1,4-dihydropyridine (1,4-DHP) derivatives with various lengths of the alkyl chain at positions 3 and 5 of the 1,4-DHP ring were studied earlier as membranotropic compounds. These amphiphiles were found to condense and efficiently deliver plasmid DNA (pDNA) into different cell lines in vitro [23,24]. It was demonstrated that dodecyloxycarbonyl substituents at positions 3 and 5 of the 1,4-DHP molecule were optimal for gene transfection efficacy in the group of these synthetic lipid-like amphiphiles [23].

1,4-DHPs are important heterocyclic scaffolds with exceptional biological properties, and they take an important position in synthetic, medicinal and bioorganic chemistry [25]. Representatives of 4-aryl-1,4-DHPs are known as calcium channel blockers and have been widely used for the treatment of hypertension [26]. After the discovery of excellent therapeutic benefits of 1,4-DHP derivatives as calcium antagonists, the number of other activities of 1,4-DHPs, such as neuroprotective [27], radioprotective [28], antimutagenic [29], antioxidative [30], anticancer [31] and antimicrobial [32,33] have been reported. 

Over the last 20 years, studies of pyridinium moieties containing compounds based on a 1,4-DHP core have revealed that they possess a number of unique properties. Cationic amphiphiles derived from polyfunctional 1,4-DHPs possess self-assembling properties that are sufficient to form nanoaggregates spontaneously without surfactants in an aqueous environment as lipid-like compounds due to the presence of both hydrophobic and hydrophilic parts in the molecule. These compounds based on the 1,4-DHP core are attractive because, along with self-assembling properties, they contain 1,4-DHP as an active linker [34,35], which is an intrinsic structural part of many pharmacologically active compounds and drugs with highly specific physiological activities (cardiovascular, anticancer, antimutagenic, as L-type calcium channel blockers, etc.) [36,37]. Additionally, these compounds have been reported to possess antiradical [38], and in vitro cell growth modulating activities [39]. Derivatives with N-dodecylpyridinium or N-hexadecylpyridinium moiety at the 1,4-DHP cycle exhibit cytotoxicity on tumor cell lines [3]. 4-(N-Dodecylpyridinium)-1,4-DHP has been reported to efficiently cross the blood–brain barrier and improve memory by enhancing the GABAergic and synapticplasticity processes [40]. Liposomes formed by these 1,4-DHPs are a promising tool for the delivery of DNA into cells [23,38]. 

The main goal of this work was to evaluate the influence of 1,4-dihydropyridine substituents on amphiphile physicochemical properties and the formation of magnetoliposomes (MLs). The following physicochemical parameters such as thermal behavior (TGA/DTA and DSC) of new amphiphiles and properties of monolayers composed by tested amphiphiles were studied. Preparation of magnetoliposomes in this study is a method to evaluate the influence of the 1,4-DHP structure on ML formation. The reverse-phase evaporation (REV) method along with the use of 1,4-DHP amphiphiles has proved its applicability to produce MLs [41,42,43]. The obtained results may add knowledge for the further comprehension of the structure–activity and liposome parameter relationships of these tested compounds.

## 2. Materials and Methods

### 2.1. Chemicals

All chemical reagents for the synthesis of the lipid-like 1,4-DHP amphiphiles were purchased from Acros Organics (Geel, Belgium), Sigma-Aldrich/Merck KGaA (Darmstadt, Germany), or Alfa Aesar (Lancashire, UK) and used without further purification. For the production of a ferrofluid containing maghemite (γ-Fe_2_O_3_) nanoparticles and iron salts (Fluka/Merck KGaA (Darmstadt, Germany), namely FeCl_2_∙4H_2_O, FeCl_3_∙6H_2_O and Fe(NO_3_)_3_, as well as nitric acid and ammonium hydroxide (Scharlau Chemie S.A., Barcelona, Spain) were used.

### 2.2. *Magnetic Nanoparticles Synthesis and Characterization*

Magnetic nanoparticles were synthesized following the Massart method [44] by co-precipitation of anionic magnetite (Fe_3_O_4_) from aqueous solutions of Fe^2+^ and Fe^3+^ chlorides using ammonium hydroxide with the following oxidation of Fe_3_O_4_ with Fe(NO_3_)_3_. As a result, positively charged γ-Fe_2_O_3_ MNPs were produced. An acidic ferrofluid (FF) was produced by peptizing the collected MNPs in an aqueous medium. To produce FF–citr with a pH~6.4, obtained γ-Fe_2_O_3_ MNPs were coated by citrate ions [45]. Trisodium citrate dihydrate was used to stabilize the magnetic nanoparticles. 

The magnetic characteristics and size distribution of the synthesized nanoparticles were determined using a vibrating sample magnetometer (Lake Shore Cryotronics, Inc., model 7404 VSM, Westerville, OH, USA) and the software for processing the magnetization data. 

### 2.3. *Synthesis of 1,4-DHP Amphiphiles*

1,1′-[(3,5-Didodecyloxycarbonyl-4-phenyl-1,4-dihydropyridine-2,6-diyl)dimethylen]bispyridinium dibromides (**1**, **4**–**6**) and 1,1′-[(3,5-dialkoxycar-bonyl-4-phenyl-1,4-dihydropyridine-2,6-diyl) dimethylen]bispyridinium dibromides (**2**, **3**) were synthesized by the previously reported methods [15,24].

1,4-DHP derivative **7** (1′-[(3,5-didodecyloxycarbonyl-4-(2-napthyl)-1,4-dihyd-ropyridine-2,6-diyl)dimethylen]bispyridinium dibromide) was synthesized in analogy with other compounds. Briefly, the developed synthesis of the cationic 1,4-DHP **7** includes three sequential steps. The first step is the synthesis of corresponding 2,6-dimethyl 1,4-DHP derivative in a two-component Hantzsch-type cyclization; the second step involves the bromination of the methyl groups of 2,6-dimethyl-1,4-DHP derivative with N-bromosuccinimide; and the third step is the nucleophilic substitution of bromine of 2,6-dibromomethylene-1,4-DHP with pyridine yielding the target compound **7**. The synthesis and characterization of the original compounds are presented in more detail in Supplementary data.

Purities of the compounds were analyzed by HPLC using the Waters Alliance 2695 system and Waters 2485 UV/Vis detector equipped with a SymmetryShield RP_18_ column (5 μm, 4.6 × 150 mm, Waters corporation, Milford, Massachusetts, USA) for parent 1,4-DHP or an Alltima CN column (5 μm, 4.6 × 150 mm, Grace, Columbia, MD, USA) for cationic moieties containing 1,4-DHP amphiphiles **1**–**7** using a gradient elution with acetonitrile/water containing 0.1% phosphoric acid as the mobile phase (*v*/*v*), at a flow rate of 1 mL/min. Peak areas were determined electronically using a Waters Empower 2 chromatography data system.

### 2.4. Thermal Analysis of 1,4-DHP Amphiphiles

#### 2.4.1. Thermogravimetric and Differential Thermal Analysis for the Tested 1,4-DHP Amphiphiles

Thermogravimetric (TGA) and differential thermal (DTA) analyses for the tested 1,4-DHP amphiphiles **1**–**7** were performed for a 3–5 mg sample with a Shimadzu DTG-60 instrument in an Ar atmosphere (Ar 5.0 from Linde Gas SIA, Riga, Latvia) with a 50 mL/min flow in a temperature range from 30 °C to 300 °C at a heating rate of 5 °C/min. Data files were transformed into an ASCII file for further analysis using TA60 ver. 2.10 software (Shimadzu Corporation, Kyoto, Japan).

#### 2.4.2. Differential Scanning Calorimetry for the Tested 1,4-DHP Amphiphiles

Dry samples of the tested 1,4-DHP amphiphiles **1**–**7** were characterized by differential scanning calorimetry (DSC). The samples were analyzed using a DSC131 evo instrument from Setaram (Caluire, France). Each sample (generally 5 to 10 mg) was weighed using an analytical scale and then cautiously placed in a 30 μL aluminum crucible. The crimped crucible was then placed in the sample compartment of a DSC instrument along with a crimped reference aluminum crucible. Argon, at a rate of 30 mL/min, was used as a purge gas. Each experiment included 3 heating-cooling cycles to determine different phase transitions. The temperature was increased at a heating rate of 10 °C/min from room temperature to approx. 130 °C depending on the sample decomposition temperature. Upon reaching the target temperature, the system was allowed to cool down to 50 °C, and an additional 2 cycles were performed in a similar way. The setup of the experiments and the data obtained were analyzed using the Calisto Data Acquisition software, ver. 1.493.

### 2.5. Characterization of Monolayers Formed by 1,4-DHP Amphiphiles or Surface Pressure–Area (π–A) Isotherms

The properties of monolayers composed of 1,4-DHP amphiphiles and their polar head areas were determined from π–*A* isotherms, which were obtained using the Langmuir–Blodgett trough. The surface pressure–molecular area (π–*A*) compression isotherms were measured using a computer-controlled Langmuir trough (Medium trough, KSV NIMA Instruments, Finland; A_total_ = 243 cm^−2^) made of Teflon and equipped with two compression barriers. The surface pressure of the monolayer was monitored with a Wilhelmy plate made of platinum, which was cleaned by flushing it with ethanol and Milli-Q water, and then burned by a Bunsen burner.

Prior to measurements, the trough and barriers were thoroughly rinsed with ethanol and Milli-Q water. Cleanliness of the aqueous surface was ensured by sweeping the barriers across the surface, and the aqueous surface was considered clean when π ≤ 0.1 mN/m. Monolayers were formed by carefully spreading an appropriate volume of the lipid solution in chloroform dropwise on the deionized water surface at 23 ± 1 °C using a Hamilton micro-syringe. The carrying solvent (CHCl_3_) was allowed to evaporate for 10 min before compressions began. The monolayers were compressed at a constant rate of 10 mm/min. Measurements were made at 23 ± 1 °C and repeated at least three times to ensure the reproducibility of the results. The experimentally detected standard deviations of the molecular area and surface pressure did not exceed 2%.

### 2.6. Magnetoliposome Preparation

To produce MLs by the REV method, the first step is to obtain of an organic phase emulsion containing a fixed amount of 1,4-DHP amphiphile as a chloroform solution, diethyl ether (3 mL) and ferrofluid (FF–citr, pH~6.4) (1 mL). This mixture was sonicated in an ultrasonic bath (Sonorex Type RK-100, Bandelin electronic GmbH, Berlin, Germany) for 20 min. Then the organic solvents were evaporated under reduced pressure (350–400 mBar) using a rotavapor (Büchi 215/V-700, Büchi Labortechnik AG, Flawil, Switzerland) and bath temperature around 30 °C. After the removal of the most of the organic solvent, viscous gel was formed, which became an aqueous suspension. Then 3 mL of deionized H_2_O was added after which a resulting mixture was evaporated under reduced pressure in the same conditions for an additional 20 min to remove traces of the solvent. The obtained suspension was filtered through a 0.45 μm syringe filter and purified by magnetic decantation to remove all non-encapsulated magnetic nanoparticles.

### 2.7. Characterization of Liposomes by Dynamic Light Scattering (DLS) and Transmission Electron Microscopy

DLS measurements of the particle hydrodynamic size distributions in the aqueous medium formed by the examined amphiphiles were performed by a Zetasizer Nano ZS instrument (Malvern Instruments Ltd., Malvern, UK) with Malvern Instruments Ltd. Software 7.12. Nanoparticles were analyzed with the following specifications: medium, water; refractive index: 1.330; viscosity: 0.8872 cP; temperature, 25 °C; dielectric constant, 78.5. Nanoparticles: liposomes; refractive index of materials: 1.60. Detection angle was 173°, with a wavelength of 633 nm. The data were analyzed using the multimodal number distribution software equipped with the instrument. The measurements were repeated three times in order to check their reproducibility.

For transmission electron microscopy studies, one drop of the sample was adsorbed to a formvar carbon-coated copper grid and negatively stained with 1% aqueous solution of uranyl acetate. The grids were examined with a JEM-1230 TEM (Jeol, Tokyo, Japan) at 100 kV.

## 3. Results and Discussion

### 3.1. *Magnetic Nanoparticle Synthesis and Characterization*

**Figure 1:**
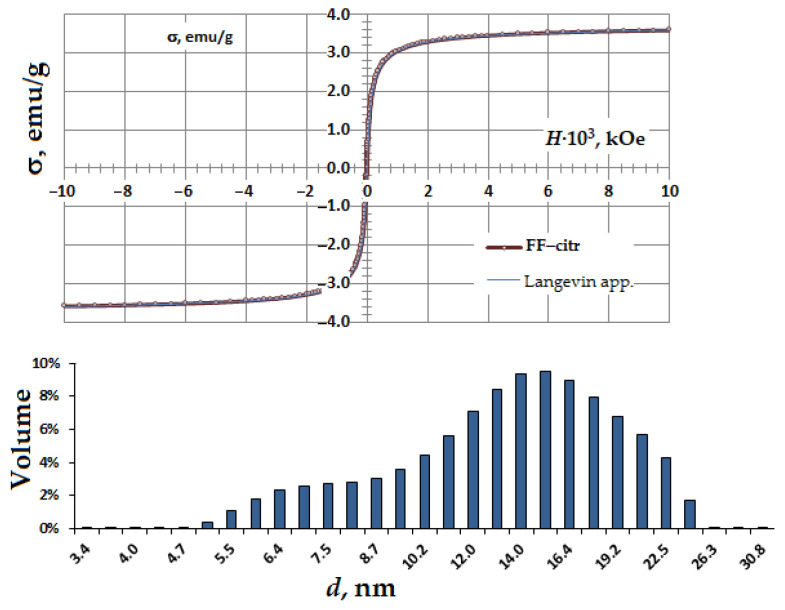
Magnetization curve and size distribution histogram of the obtained superparamagnetic γ-Fe_2_O_3_–citr magnetic nanoparticles.

Magnetic nanoparticles were synthesized by co-precipitating anionic magnetite (Fe_3_O_4_) from aqueous solutions of Fe^2+^ and Fe^3+^ chlorides using ammonium hydroxide with the following oxidation of Fe_3_O_4_ with Fe(NO_3_)_3_. As a result, positively charged γ-Fe_2_O_3_ MNPs were produced. Ferrofluid containing γ-Fe_2_O_3_–citr was obtained with an MNP coating by citrate ions, FF–citr pH~6.4. The polydispersity index of the obtained FF–citr was 0.181 ± 0.006; the ζ-potential was −38.0 ± 2.3 mV according to DLS data. The volume fraction Φ_FF–citr_ = 1.5% was determined by iron concentration colorimetric analysis (with 5-sulfosalicilic acid dehydrate, absorbance at wavelength λ = 425 nm). Fe concentration in the obtained FF–citr was 0.95 M, which corresponded to the γ-Fe_2_O_3_ nanoparticle content (76 mg/mL), and the FF density was determined to be 1.06 g/cm^3^. The magnetization curve and size distribution of the FF–citr nanoparticles are shown in the Figure 1. Magnetic properties of the synthesized MNPs were determined using a vibrating sample magnetometer and software for processing magnetization curves. The magnetic diameter of the nanoparticles in the main population was determined to be 15 nm by adjusting the magnetization curve of the ferrofluid to a Langevin formalism weighted by the size distribution of the γ-Fe_2_O_3_–citr MNPs.

### 3.2. Synthesis of 1,4-DHP Derivatives

**Figure 2:**
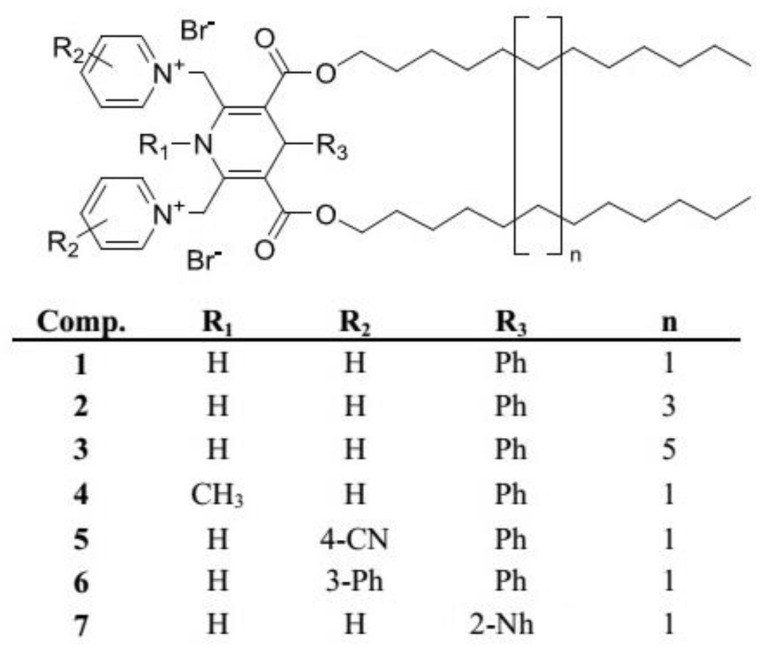
Structures of the studied 1,4-DHP amphiphiles **1**–**7**.

The synthesis of the selected 1,4-DHP amphiphiles **1**–**7** varying in substituents at the 1,4-DHP ring was carried out by the previously described methods [23,38]. The studied 1,4-DHP derivatives were divided into four groups to evaluate the influence of the structure elements on the physicochemical properties of the compounds and on the properties of MLs (Figure 2):

- 1,4-DHPs with different alkyl chain lengths at 3,5-ester moieties of 1,4-DHP (C_12_, C_14_ and C_16_) (comps. **1**–**3**);
- variation of the N-substituent at position 1 of 1,4-DHP (N-H or N-CH_3_) (comps. **1** and **4**);
- variation of the substituents at pyridinium moieties as cationic head groups at positions 2 and 6 of 1,4-DHP (H, 4-CN and 3-Ph) (comps. **1**, **5** and **6**);
- variation of the substituent at position 4 of 1,4-DHP (Ph and Nh) (comps. **1** and **7**).

1,1′-[(3,5-Bisdodecyloxycarbonyl-4-phenyl-1,4-dihydropyridine-2,6-diyl)dimethylen]bis(pyridin-1-ium) (or substituted pyridinium) dibromides (**1**, **5**, **6**), 1,1′-[(3,5-dialkoxycar-bonyl-4-phenyl-1,4-dihydropyridine-2,6-diyl)dimethylen]bis(pyridine-1-ium) dibromides (**2**, **3**) and 1,1′-[(3,5-didodecyloxy-carbonyl-4-(2-napthyl)-1,4-dihydropyridine-2,6-diyl)dimethylen]bis(pyridin-1-ium) dibromide (**7**) were obtained according to Scheme S1 (Supplementary data). Briefly, the corresponding parents 3,5-bis(alkoxycarbonyl)-2,6-dimethyl-4-aryl-1,4-dihydropyridines were obtained by the classical Hantzsch synthesis from the corresponding acetoacetic ester, the corresponding aldehyde and ammonium acetate [38].

1,1′-[(3,5-Bis((dodecyloxy)carbonyl)-1-methyl-4-phenyl-1,4-dihydropyridine-2,6-di-yl)dimethylene]-bis(pyridin-1-ium) dibromide (**4**) was obtained according to Scheme S2 (see the Supplementary data). Briefly, the parent 3,5-didodecyloxy-carbonyl-4-phenyl-1,2,6-trimethyl-1,4-dihydropyridine was synthesized from dodecyl acetoacetate, benzaldehyde and methylamine hydrochloride as a nitrogen source in pyridine by refluxing the reaction mixture for 6 h. 

Bromination of 2,6-methyl groups of parent 1,4-DHP was performed by N-bromosuccinimide in methanol giving 2,6-di(bromomethyl)-3,5-bis(alkoxy-carbonyl)-4-aryl-1,4-dihydropyridine, which without purification was treated by the corresponding pyridine derivative resulting in formation of the target 1,4-DHP amphiphiles **1**–**7**.

^1^H-NMR spectra data and other physicochemical parameters of compounds **1**–**6** were in agreement with those reported in the literature [23,38,46]. Characterization of the original compounds—1,4-dihydropyridine (1,4-DHP) amphiphiles **1**–**3**, **5**–**7**—is given in the Supplementary data. Measured by LC–MS mass-to-charge (m/z) values of the re-synthesized compounds were in good agreement with the calculated values and also with the previously reported ones. In addition, the characteristic signals of 2,6-methylene group protons in ^1^H-NMR spectra were observed as an AB-system, which confirmed the diastereotopic properties of CH_2_X protons in the molecules of 1,4-DHP amphiphiles and confirmed their structures [38]. The purities of the studied compounds were at least 98% according to high-performance liquid chromatography data.

It is known from the literature that some 1,4-DHP molecules exhibit a significant sensitivity to light, leading to the complete loss of pharmacological activity [47,48]. It is necessary to emphasize that the tested cationic moieties containing 1,4-DHP are more stable than the corresponding parent compounds without cationic moieties. Our previous studies of electrochemical oxidation of 1,4-DHP derivatives containing cationic pyridinium methylene groups in position 2 and 6, by cyclic voltammetry on a stationary glassy carbon electrode in dry acetonitrile, demonstrated that they had electrooxidation potentials of 1.57–1.58 V [49]. These data were also in agreement with our previous results, where the electrochemical oxidation potential of a similar cationic 1,4-DHP was determined as 1.7 V, and the electrochemical oxidation of this compound was characterized as a two-electron process [50], whereas the parent compounds—1,4-DHP derivatives without cationic moieties demonstrated lower electrooxidation potentials. Thus, 4-phenyl substituted Hantzsch 1,4-dihydropyridine had a potential of 1.08 V [51], and other different 4-aryl substituted 1,4-DHPs had potentials around 1.1 V [52], but 4-monoalkyl substituted 1,4-DHPs had oxidation potentials of 1.01–1.03 V, respectively [53]. 

Additionally, it was demonstrated that pyridinium moieties containing 3,5-didodecyloxycarbonyl-4-phenyl-1,4-dihydropyridine derivatives showed 25–60% radical scavenging activity, which was comparable with the antiradical activity (ARA) of Diludin (40%)—a widely known antioxidant. Other 1,4-DHP amphiphiles containing saturated heterocyclic moieties—N-methylmorpholinium or N-methylpyrrolidinium derivatives—demonstrated more pronounced ARA, namely 95% and 54%, respectively [38]. The choice of cationic moiety containing 1,4-DHP amphiphiles **1**–**7** for the evaluation of their physicochemical properties in order to determine stable and safe lipids for formation of magnetoliposomes is based on the above mentioned data.

### 3.3. Thermal Analysis of 1,4-DHP Amphiphiles

It is known that the temperature of phase transition depends on the structure of the hydrocarbon chains in lipid molecules and also on the nature of their polar heads. 1,4-DHP amphiphiles **1**–**7** were tested using the TGA/DTA technique to determine the thermal stability and phase transitions of the compounds. Along with the TGA/DTA technique, the compounds also were tested by DSC to clarify in detail phase transitions that occur before the decomposition of the substance starts. Thermal studies were carried out in order to assess how the structures of the amphiphiles influence their thermal stability and phase transition. The values obtained by analyzing the TGA/DTA curves are presented in Table S1 (Supplementary data).

**Figure 3:**
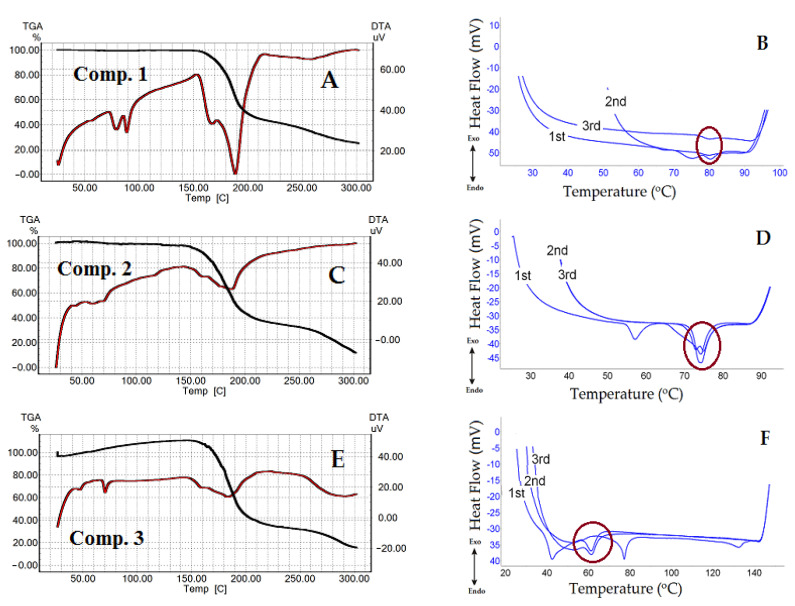
(**A**,**C**,**E**): TGA/DTA curves for comps. **1**–**3**. (**B**,**D**,**F**): DSC triple heating curves of comps. **1**–**3** in the temperature ranges before compound decomposition. The circles show endothermic peaks reproduced during the three heating cycles.

**Figure 4:**
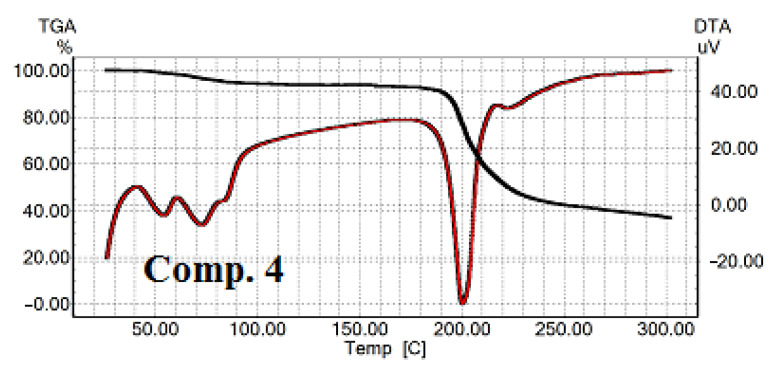
TGA/DTA curves for comp. **4**.

**Figure 5:**
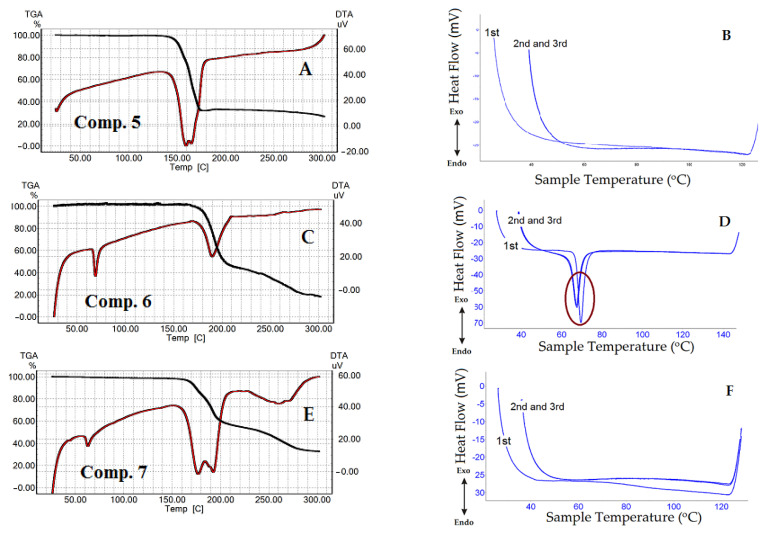
(**A**,**C**,**E**): TGA/DTA curves for comps. **5**–**7**. (**B**,**D**,**F**): DSC triple heating curves of comps. **5**–**7** in the temperature range before compound decomposition. Endothermic peaks that occurred after triple heating are marked by circles.

As shown in Figure 3A,C,E, Figure 4 and Figure 5A,C,E, the last transitions of all compounds correspond to the compounds’ decomposition. Analysis of the TGA curves of the samples and comparison with the DTA curves show that when approaching the temperatures of the last transition, the sample noticeably started losing weight, which means the beginning of the compound decomposition process. The decomposition process is accompanied by significant heat absorption. Figure 3A,C,E shows the dynamics of comps. **1**–**3** curves as a function of the heating temperature obtained by the TGA/DTA technique. The curve of comp. **1** exhibited one weak and two distinct endothermic peaks (Figure 3A). The first transition temperature peak for comp. **1** (Figure 3A) was at 56 °C, but it had low intensity. TGA data for comp. **1** were in a good agreement with our previously published results [54]. With an increase in the length of the lipophilic chains, “broadening” of the peaks was observed (Figure 3C,E, comps. **2** and **3** and Table S1). It should be admitted that N-CH_3_-substituted 1,4-DHP showed a similar trajectory of the DTA curves (Figure 4 and Table S1) as unsubstituted 1,4-DHP. Both compounds had a distinct first order endothermic transition, but the transition of comp. **4** had a wider temperature range, the so-called “broadening” transition.

In Figure 3B,D,F, united curves of comps. **1**–**3** after triple heating obtained by the DSC technique are shown. The samples of the 1,4-DHP amphiphiles to be analyzed by DSC technique were heated up to temperatures below the compound decomposition temperatures, which were determined by TGA for each substance minus 10–20 °C. This process was repeated three times to examine also the thermal stability of the compounds and the repeatability trajectory of the curves. The DSC curves for comps. **1**–**3** demonstrated common features: the curves at the first heating showed several transitions, but the curves at the second and third heating displayed only one transition. The results of the heating and cooling curves analysis are listed in Table 1. In addition, Table 1 lists the results of sample cooling (exothermic processes), which correspond to those of the endothermic process when the compound was heated.

The curves of compounds **1**–**3** and **6** demonstrated exothermic peaks after triple heating and cooling. For comps. **1** and **2**, the exothermic process showed weak signals (see the cooling curves in Figure S1 in the Supplementary data).

TGA/DTA analysis showed that comp. **5** (4-CN substituent in the pyridinium at cationic head groups) has no endothermic transition (Figure 5A) at a temperature lower than the compound decomposition temperature. This fact was confirmed by DSC (Figure 5B). Comp. **6** (3-Ph substituent at cationic head groups) had a pronounced endothermic peak, which was observed in the same temperature range in the DSC curves (Figure 5C for DTA and Figure 5D for DSC curves). A comparison of comp. **5** and comp. **6** makes it possible to observe the effect of the substituent on the thermal behavior and thermal stability of these compounds. Variation of the substituent at position 4 of 1,4-DHP, namely phenyl for comp. **1** and napthyl for comp. **7**, also confirmed this influence (Figure 3A and Figure 5E). The curve of comp. **7** showed a rather narrow first order endothermic transition temperature range when tested by TGA/DTA; this first transition had a rather small absorbed heat value of 57.75 J/g. However, this transition was not confirmed by DSC for comp. **7** (Figure 4F).

It is underlined in the literature that the properties of the liposomes are mainly dependent on the physicochemical characteristics of lipids. It is known that the length and the degree of saturation of the lipid chain influence the phase transition temperature, such as gel to liquid crystalline state. The phase transition temperature depends on the length of the fatty acid chains, their degree of saturation, charge and head group types [55,56]. Usually a longer alkyl chain has a higher transition temperature, and introduction of double bonds decreases the transition temperature [56,57,58]. All studied 1,4-DHP amphiphiles have saturated lipophilic chains. For comps. **1**–**3** with increasing length of alkyl chains for two CH_2_ groups, the values of the first transition temperature according to TGA data were in the same range of 56 °C, 44 °C and 54 °C (Table S1), respectively. This could be explained by the influence of pyridinium as cationic head group or 1,4-DHP core as an active linker. Our previous data regarding thermogravimetric analysis of structurally related pyridine amphiphiles with various heterocycles as cationic head groups demonstrated a wider transition phase range for the first transition state [54]. In agreement with DSC data, these temperatures for comps. **1**–**3** were 80 °C, 75 °C and 62 °C, respectively. Similar phenomena were observed for liposomal compositions of DPPC with monocationic 1,4-DHP amphiphiles—4-(N-alkylpyridinium-1,4-DHP)—as additives where with the increase of alkyl chain length, the phase transition temperature of compositions was decreased [59].

### 3.4. Surface Pressure–Area Isotherms, Mechanical Properties of Monolayers

Surface pressure (π) is defined as the decrease in surface tension of the aqueous medium when surfactant is added, $\pi =\gamma_{0}-\gamma$, where γ_0_ is the surface tension of water and γ is the surface tension of water with the surfactant monolayer. As seen from the π−*A* isotherms, all studied compounds were able to form stable monolayers in an aqueous medium. Graphs of the surface pressure for monolayers composed of 1,4-DHP amphiphiles **1**–**7** were plotted. The critical surface pressure is defined as the surface pressure at which the monolayer collapses. 

As is shown in Figure S2 (Supplementary data), all compounds had a similar collapsing surface pressure around 45 mN/m (Table 2) except for comp. **5** (4-CN), which had a critical pressure of 53 mN/m. All of the studied compounds had a similar π−*A* isotherm pattern without an apparent liquid expanded (LE) to liquid condensed (LC) phase transition.

Compressibility moduli $\;C_{s}^{-1}$, mN/m (Figure S3, Supplementary data) were calculated for comps. **1**–**7** (Table 2) from the π−*A* data obtained from the monolayer compressions using the following Equation (1) [60]:

(1) $$C_{s}^{-1}=-A\;\left(\delta \pi /\delta A\right)$$

where δπ/δA is the slope of the monolayer, and the area, *A*, corresponds to the mean molecular area (MMA) at the indicated surface pressure, π. According to the literature [60], the values of the compressibility modulus ranging from 0 to 12.5 mN/m refer to the gas phase of the films, from 12.5 to 50 mN/m for the liquid-expanded (LE) films, from 100 to 250 mN/m for the liquid-condensed (LC) films, and above 250 mN/m for the solid films (S) [60,61].

According to the values of $C_{s}^{-1},\;$ the monolayers collapse in the LC phase, and none of the compounds reach the solid phase. A pronounced decrease in $C_{s}^{-1}\;$ occurs at a surface pressure about 5–8 mN/m below the critical pressure acquired from the π–A isotherms, which means that the collapse of the monolayer does not spontaneously happen at the critical pressure point, but starts earlier, which is in accordance with a different behavior of the surfactant monolayers [38].

The MMA of the compound of interest can be extracted from the π−*A* isotherms in the liquid condensed phase, and the obtained values for comps. **1**–**7** are listed in Table 3. It is a matter of long debate whether the hydrophobic tail has an influence on the surfactant area per molecule or not. Obtained results demonstrate that the alkyl chain length in the ester moieties varying from C_12_ to C_16_ (comps. **1**, **2** and **3**) did not significantly influence the mean molecular area. On the other hand, the methylation of the dihydropyridine nitrogen atom at position 1 enlarged the area of the molecule almost by 15%. As could be expected, the introduction of a large hydrophobic phenyl group to pyridinium moieties in the positions 2 and 6 of the 1,4-DHP cycle enlarged the MMA, but a relatively small cyano group did not. In contrast, the introduction of a bulky naphthyl moiety in position 4 of the 1,4-DHP core slightly decreased the MMA. This means that the tail length and moieties in the position 4 of 1,4-DHP are not important in terms of the MMA, in contrast to the 1,4-DHP N-substituents and hydrophobic substituents at the pyridinium moiety as the cationic part of the amphiphile.

The packing parameter of compounds **1**–**7** was calculated using the obtained molecular areas (see Table 3) from the following Equation (2) [62,63]:

(2) $$p=v_{0}/al_{0},$$

where $v_{0}$ and $l_{0}$ are the volume and the length of the surfactant tail [62], and a is the equilibrium area per molecule. The packing parameter values determine the shape of the micelle formed in the aqueous medium (*p* = 0 ≤ 1/3 for the sphere, 1/3 ≤ 1/2 for the cylinder, and 1/2 ≤ 1 for the flexible bilayer) vesicles [62,63]. Every studied compound had *p* values near 1/2 or higher (see Table 3), which led to an assumption that comps. **1**–**7** form bilayer structures in the aqueous medium. According to the elaborated theoretical considerations, the formation of vesicles occurs in the systems with the packing parameters between 1/2 and 1 [63].

### 3.5. Magnetoliposome Preparation, Evaluation and Characterization

**Figure 6:**
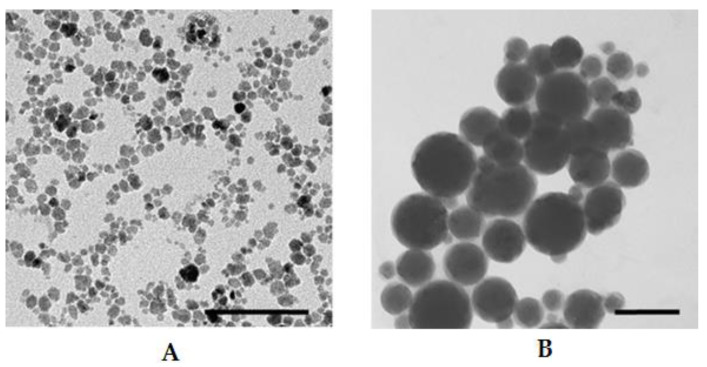
TEM images of (**A**) γ-Fe_2_O_3_ nanoparticles and (**B**) magnetoliposomes (MLs) formed by magnetic nanoparticles (MNPs) coated with citrate ions (γ-Fe_2_O_3_–citr 55 mg/mL) and 1,4-DHP **1**; n_DHP_/n_γ-Fe2O3_ = 0.04. Scale bar is 100 nm. MLs obtained by the reverse-phase evaporation (REV) method.

Cationic moiety containing 1,4-DHP amphiphiles **1**–**7** were chosen as lipid-like compounds for evaluation to produce magnetoliposomes (MLs) and characterization of liposome properties. According to our previous work [38], comp. **1** displays more pronounced delivery activity. Therefore we used this compound as the standard for characterization of other amphiphiles. The MLs shown in Figure 6B were obtained by the REV method from 1,4-DHP amphiphile **1** and FF containing negatively charged MNPs coated by citrate anions (γ-Fe_2_O_3_-citr) with pH~6.4 shown in Figure 6A. Transmission electron microscopy images (Figure 6) showed MLs with diameters in the range of 50–100 nm, while diameters of pure MNPs were around 10–15 nm (see MNP size distributions in Figure 1 and the TEM image in Figure 6A). It was confirmed that 1,4-DHP amphiphile **1** and MNPs coated with citrate anions formed magnetoliposomes with sizes that are applicable in biomedicine purposes.

For further studies, 1,4-DHP amphiphiles **1**–**3** and **7** were chosen. Initially “empty” liposomes of pure 1,4-DHP amphiphiles **1**–**3** and **7** without FF additive were prepared by the REV method. DLS data of “empty” liposomal samples are summarized in Table 4 and Figure S4 in the Supplementary data.

The results for comp. **1** confirmed that the sizes of liposomes prepared by SpSw and REV methods were comparable. The dispersions for comp. **1** prepared by both the REV and SpSw methods had the lowest PdI value of 0.263, which indicated that the samples were more homogeneous than those of the other compounds, while the other samples were identified as moderately homogeneous samples, with PdI values of 0.428–0.486. DLS data analysis showed that the average diameter of the “empty” liposomes formed by amphiphiles **1**–**3** and **7** were in the 107–291 nm range. All the tested amphiphiles formed mainly one liposome population, namely 93–97% for comps. **1**, **3**, and **7** and 79% for comp. **2**.

For detailed studies, 1,4-DHP amphiphiles **1**–**3** and **7** were chosen as membrane-forming agents to produce magnetoliposomes by the reverse-phase evaporation (REV) method. After encapsulation, the mixture was purified by magnetic decantation to remove all non-encapsulated MNPs. As underlined in the literature for similar liposomal systems, considering the strong difference in maximum magnetization of magnetic nanoparticles and magnetoliposomes in aqueous media, due to the diamagnetic contribution of water, the aqueous magnetoliposomes are not attracted to the magnet. Therefore, only non-encapsulated magnetic nanoparticles are separated in this way, keeping the magnetoliposomes in the supernatant phase. The lipid phase remains unchanged upon decantation, with the initial and final lipid concentrations being the same in the sample [64].

The total iron content encapsulated in MLs was around 20% from the starting concentration, which was confirmed by iron detection calorimetric analysis of the magnetoliposomal samples obtained by REV. The DLS data of magnetoliposomal samples are summarized in Table 5 and Figure S5 in the Supplementary data.

The obtained results demonstrated that main characteristic parameters for liposomal samples were comparable for “empty” liposomes and MLs. PdI values for MLs samples of comps. **1**–**3** were 0.246–0.291, indicating homogeneity of samples, while the PdI value of the sample of comp. **7** was 0.521. DLS data analysis showed that the average diameter of the MLs formed by amphiphiles **1**–**3** and **7** were in the 138–299 nm range. All tested amphiphiles formed mainly one liposome population, namely 93–99% for comps. **1**–**3** and 70% for comp. **7**.

## 4. Conclusions

Targeted modification of the 1,4-DHP core with different substituents both in the polar and in the non-polar parts of the molecule was performed, resulting in four groups of amphiphiles for the evaluation of the influence of structural elements on the physicochemical properties of compounds and on the properties of magnetoliposomes. 

The obtained results by TGA/DTA demonstrate that with increasing length of the ester chains for 1,4-DHP amphiphiles **1**–**3**, the transition temperatures were shifted to lower temperatures. By TGA/DTA, transition temperatures are first transition, 56 °C; and second transition, 79 °C for comp.**1**; first transition, 44 °C; and second transition, 60 °C for comp. **2**; and first transition, 54 °C; and second transition, 64 °C for comp. **3**. By DSC, these temperatures were 80 °C (comp. **1**), 75 °C (comp. **2**) and 62 °C (comp. **3**). Due to the relatively higher phase transition temperature, these amphiphiles may be potentially used as additives in composition with other synthetic lipids for the development of nanovectors.

It was shown that the variation of the alkyl chain length or the change of substituents at position 4 of 1,4-DHP did not show a significant influence on the mean molecular areas of the tested compound monolayers. In contrast, the introduction of the N-methyl substituent at position 1 of the 1,4-DHP molecule enlarges the mean molecular area almost by 15%, and also an addition of hydrophobic sterically hindered phenyl substituents at pyridinium moieties at positions 2 and 6 of the 1,4-DHP molecule slightly decreases the mean molecular area of the compound. The transition to the LC phase of comps. **1**–**7** is not clearly distinguished and occurred at 20–25 mN/m. Additionally, it was suggested that the tested 1,4-DHP amphiphiles **1**–**7** form bilayer structures in the aqueous medium because the calculated packing parameter values range from 0.43 to 0.55, which is in agreement with the theoretical considerations that the formation of vesicles occurs in the systems with the packing parameters between 1/2 and 1 [62].

It was demonstrated that the variation of the alkyl chain length or the change of substituents at position 4 of 1,4-DHP did not show a significant influence on the properties of liposomes.

## Figures and Tables

**Table 1:** Values obtained from DSC curves. “↓“ denotes endothermic and “↑“ denotes exothermic transition. Compounds **5** and **7** did not show any transitions before the decomposition temperature. Cooling curves are presented in the Supplementary data (Figure S2).

Comp.	Transitions Temperatures Range, °C				
	Transition	1st Heating (Process)	2nd Heating (Process)	3rd Heating (Process)	All Coolings (Process)
**1**	1st 2nd 3rd	65.22–70.05 ↓ 70.05–78.08 ↓ 78.10–82.70 ↓	77.21–82.96 ↓	77.00–82.70 ↓	65.00–60.00 (weak signal)
**2**	1st 2nd	55.20–60.15 ↓ 69.90–76.42 ↓	71.97–76.83 ↓	70.00–78.00 ↓	75.27–72.02 ↑
**3**	1st 2nd	37.31–57.42 ↓ 70.86–81.57 ↓	57.42–66.44 ↓	56.82–66.71 ↓	63.53–55.89 (weak signal)
**6**	1st	64.20–76.32 ↓	59.14–74.98 ↓	59.58–74.54 ↓	56.53–44.63 ↑

**Table 2:** Mechanical properties of monolayers composed of 1,4-DHP amphiphiles **1**–**7**. P (mN/m) is the critical pressure of the monolayers; $C_{s}^{-1}$ mN/m) is the compressibility modulus.

Comps.	1	2	3	4	5	6	7
P ± SD, mN/m	46.62 ± 0.02	46.53 ± 0.75	46.33 ± 0.58	48.53 ± 0.68	53.06 ± 0.68	44.81 ± 0.44	47.13 ± 0.74
$C_{s}^{-1}\;$± SD, mN/m	156.65 ± 1.24	170.46 ± 2.37	160.18 ± 1.58	170.44 ± 3.95	209.98 ± 1.69	120.71 ± 3.11	189.12 ± 2.92

**Table 3:** Mean molecular areas (MMA, Å^2^) of the 1,4-DHP amphiphiles **1**–**7**, and calculated values of the packing parameter (p) of these compounds.

Comps.	1	2	3	4	5	6	7
MMA ± SD, Å^2^	82.77 ± 0.60	82.48 ± 1.19	86.91 ± 1.50	95.01 ± 0.94	82.91 ± 0.61	96.64 ± 1.08	76.65 ± 1.59
*p* *	0.51	0.51	0.48	0.44	0.51	0.43	0.55

* For all compounds SD < 0.02.

**Table 4:** DLS data of “empty” liposome dispersions of 1,4-DHP amphiphiles **1**–**3** and **7** obtained by the REV method without ferrofluid (FF) additive. PdI is the polydispersity index; Z-ave D_H_ is the diameter that represents the mean hydrodynamic diameter of all liposomes in the distribution. The mean hydrodynamic diameter, D_H_, depicts the hydrodynamic size of the main population of the tested sample.

“Empty” Liposomes	PdI	Z-ave D_H_, nm	Distr. Peaks (max) Mean D_H,_ nm (%)		
			Peak 1 (%)	Peak 2 (%)	Peak 3 (%)
Comp. **1** *	0.263 ± 0.050	146.4 ± 1.4	188 (97)	4757 (3)	–
Comp. **1**	0.263 ± 0.013	150.0 ± 8.1	190 (97)	3246 (3)	–
Comp. **2**	0.486 ± 0.007	106.9 ± 8.3	216 (79)	34 (18)	4491 (3)
Comp. **3**	0.431 ± 0.013	219.2 ± 5.4	347 (95)	46 (5)	–
Comp. **7**	0.428 ± 0.017	290.6 ± 4.6	557 (93)	34 (2)	2829 (5)

* spontaneous swelling (SpSw).

**Table 5:** DLS data of liposomes of 1,4-DHP amphiphiles **1**–**3** and **7** obtained with FF–citr. PdI is the polydispersity index; Z-ave D_H_ is the diameter that represents the mean hydrodynamic diameter of all liposomes in the distribution. The mean hydrodynamic diameter, D_H_, depicts the hydrodynamic size of the main population of the tested sample. Dispersions were prepared by the REV method.

Liposomes	PdI	Z-ave D_H_, nm	Distr. Peaks (max) Mean D_H,_ nm (%)		
			Peak 1 (%)	Peak 2 (%)	Peak 3 (%)
Comp. **1**	0.291 ± 0.016	299.3 ± 1.8	432 (93)	58 (7)	–
Comp. **2**	0.246 ± 0.017	149.6 ± 1.0	204 (94)	38 (6)	–
Comp. **3**	0.286 ± 0.001	137.6 ± 0.1	201 (99)	4896 (1)	–
Comp. **7**	0.521 ± 0.003	143.8 ± 0.7	316 (70)	54 (25)	4567 (5)
FF–citr	0.181 ± 0.006	38.4. ± 0.4	47 (100)	–	–

## Data Availability

The data presented in this study are available within this article and in Supplementary Data.

## Supplementary Materials

The following are available online at https://www.mdpi.com/2079-4991/11/3/593/s1. Scheme S1. Synthesis of 1,4-dihydropyridine (1,4-DHP) amphiphiles **1**–**3**, **5**–**7**; Scheme S2. Synthesis of 1,4-dihydropyridine (1,4-DHP) amphiphile **4**; Table S1. Temperatures characteristics of tested compounds **1**–**7**, obtained by analysing TGA and DTA curves. Figure S1. Cooling curves obtained by DSC after heating process for tested compounds **1**–**3**, **5**–**7**; Figure S2. 1,4-DHP amphiphiles **1**–**7** surface pressure—mean molecular area isotherms at 23 ± 1 °C; Figure S3. Compressibility modulus-surface pressure dependences obtained for the 1,4-DHP amphiphiles **1**–**7** monolayers; Figure S4. Hydrodynamic size distribution of the ‘empty’ liposomes formed by 1,4-DHP amphiphiles **1**–**3** and **7**. Liposomes obtained by REV; Figure S5. Hydrodynamic size distribution of the magnetoliposomes formed by 1,4-DHP amphiphiles **1**–**3** and **7**.
