# Supplementary material for: Evaluation of Physicochemical Properties of Amphiphilic 1,4-Dihydropyridines and Preparation of Magnetoliposomes
Source: Nanomaterials (Basel). 2021 Feb 27;11(3):593. doi: 10.3390/nano11030593 (PMC7996955; doi:10.3390/nano11030593)
Supplement: Supplementary file 1 [file nanomaterials-11-00593-s001.pdf]

nanomaterials

*Supplementary information*

# Evaluation of Physicochemical Properties of Amphiphilic 1,4-Dihydropyridines and Preparation of Magnetoliposomes

**Oksana Petrichenko [1,*], Aiva Plotniece [2,3], Karlis Pajuste [2], Martins Rucins [2], Pavels Dimitrijevs [2,3], Arkadij Sobolev [2], Einars Sprugis [4] and Andrejs Cēbers [1]**

[1] Laboratory of Magnetic Soft Materials, Faculty of Physics, Mathematics and Optometry, University of Latvia, 3 Jelgavas str., Riga LV-1004, Latvia; andrejs.cebers@lu.lv (A.C.)
[2] Latvian Institute of Organic Synthesis, 21 Aizkraukles Str., Riga LV-1006, Latvia; aiva@osi.lv (A.P.); kpajuste@osi.lv (K.P.); rucins@osi.lv (M.R.); p.dimitrijevs@osi.lv (P.D.); arkady@osi.lv (A.S.)
[3] Department of Pharmaceutical Chemistry, Faculty of Pharmacy, Riga Stradiņš University, 21 Dzirciema Str., Riga LV-1007, Latvia
[4] Laboratory of Chemical Technologies, Institute of Solid State Physics, University of Latvia, 8 Kengaraga Str., Riga LV-1063, Latvia; esprugis@cfi.lu.lv
* Correspondence: Oksana.petricenko@lu.lv

*Synthesis of 1,4-DHP amphiphiles **1**–**7***

1,1'-[(3,5-bisdodecyloxycarbonyl-4-phenyl-1,4-dihydropyridine-2,6-diyl)dimethylen]-bispyridinium (or substituted pyridinium) dibromides **1**,**5**,**6**; 1,1'-[(3,5-dialkoxycarbonyl-4-phenyl-1,4-dihydropyridine-2,6-diyl)dimethylen]-bispyridinium dibromides **2**,**3** and 1,1'-[(3,5-didodecyloxycarbonyl-4-(2-napthyl)-1,4-dihydropyridine-2,6-diyl)dimethylen]-bispyridinium dibromide (**7**) were obtained according to Scheme S1.

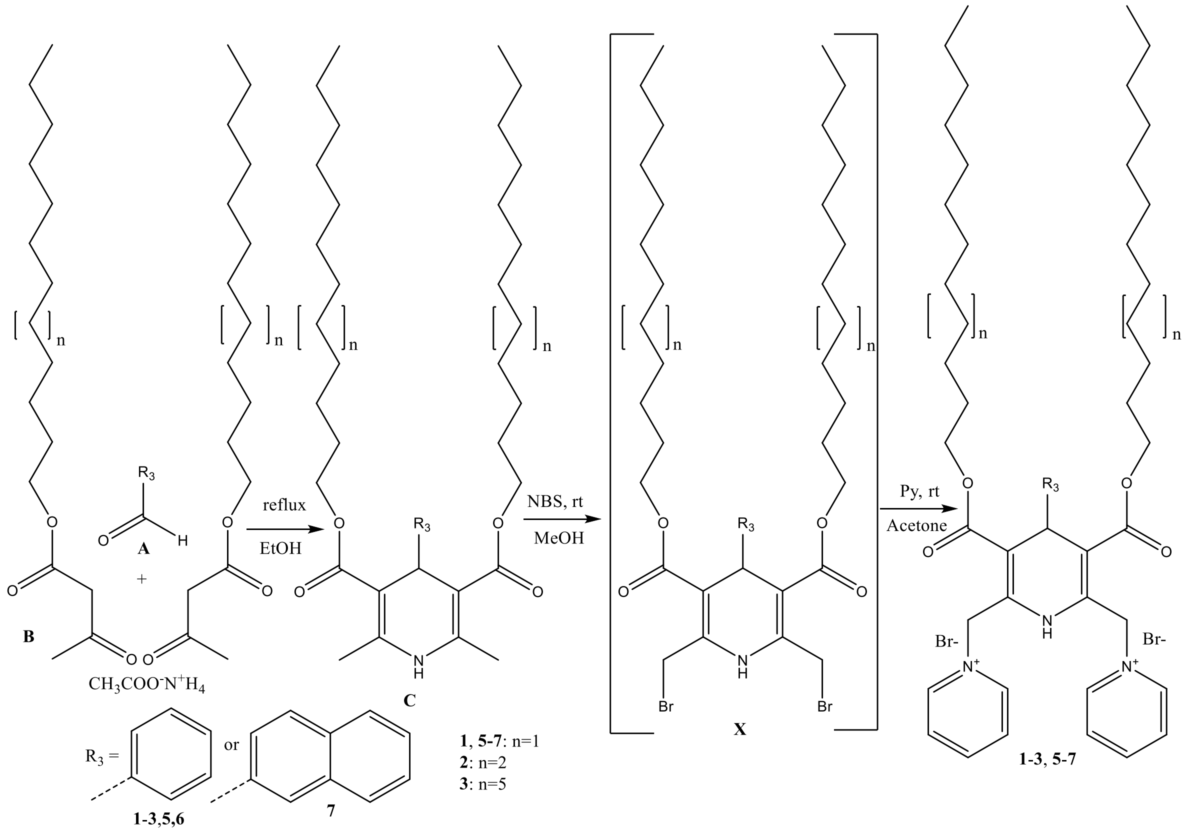


**Scheme S1.** Synthesis of 1,4-dihydropyridine (1,4-DHP) amphiphiles **1**–**3**, **5**–**7**.

1,1'-((3,5-Bis((dodecyloxy)carbonyl)-1-methyl-4-phenyl-1,4-dihydropyridine-2,6-diyl)bis(methylene))bis(pyridin-1-ium) dibromide (**4**) was obtained according to Scheme S2.

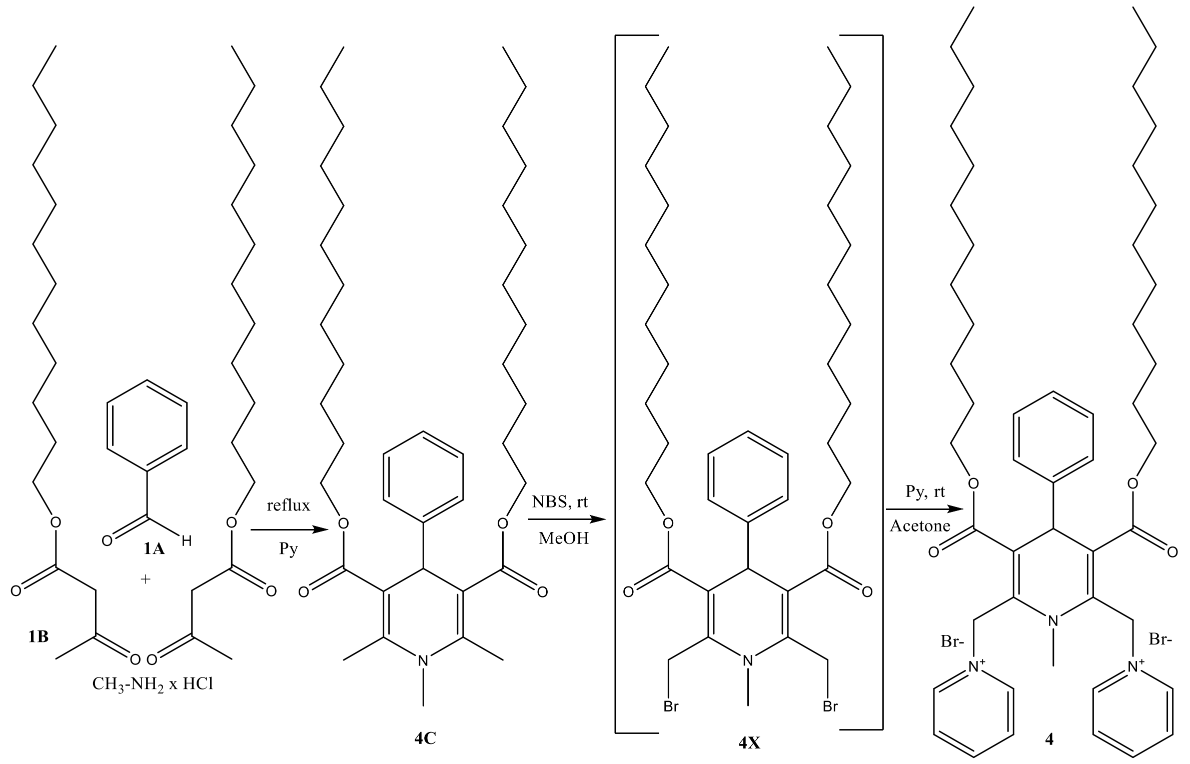


**Scheme S2.** Synthesis of 1,4-dihydropyridine (1,4-DHP) amphiphile **4**.

Briefly, the corresponding 3,5-bis(alkoxycarbonyl)-2,6-dimethyl-4-aryl-1,4-dihydropyridines (**C**) (Scheme S1) were obtained from the corresponding acetoacetic ester **B** (2eq), corresponding aldehyde **A** (1eq) and ammonium acetate (1.2 eq) in the classical Hantzsch synthesis [1,2].

The 3,5-didodecyloxycarbonyl-4-phenyl-1,2,6-trimethyl-1,4-dihydropyridine **4C** (Scheme S2) was synthesized from dodecylacetoacetate (**1B**) (2 eq), benzaldehyde (**1A**) (1eq), methylamine hydrochloride (1eq) in pyridine by refluxing a reaction mixture for 6 h [1].

Bromination of 2,6-methyl groups of 1,4-DHPs **C** were performed by N-bromosuccinimide (NBS) (2eq) in methanol giving 2,6-di(bromomethyl)-3,5-bis(alkoxycarbonyl)-4-aryl-1,4-dihydropyridines **X** which without purification were treated by the corresponding pyridine derivative (2.2 eq) at room temperature in acetone giving the target 1,4-DHP amphiphiles **1**-**7**.

$^{1}$H-NMR spectra data and other physicochemical parameters of compounds **1**-**6** were in agreement with those reported in the corresponding literature [1–3]. Measured by LC-MS mass-to-charge (m/z) values of the re-synthesized compounds were in good agreement with the calculated values and also previously reported ones. Also the characteristic signals of 2,6-methylene group protons in $^{1}$H NMR spectra were observed as an AB-system, which confirmed diastereotopic properties of $CH_2X$ protons in the molecules of 1,4-DHP amphiphiles and confirm their structure. [2]

Purities of synthesized compounds were analyzed with HPLC on Waters Alliance 2695 system and Waters 2485 UV/Vis detector at 254 nm equipped with SymmetryShieldTM RP18 column (5 µm, 4.6 x 150 mm, Waters corporation, Milford, MA, USA) using a gradient elution with acetonitrile/water containing 0.1% phosphoric acid as the mobile phase (v/v), at a

flow rate of 1 mL/min. Peak areas were determined electronically with Waters Empower 2 chromatography data system. Studied compounds **1–7** were at least 98% according to high performance liquid chromatography (HPLC) data.

*Conditions for HPLC analysis of parent 1,4-DHP* ***C****.*

Synthesized compounds were analyzed with HPLC on Waters Alliance 2695 system and Waters 2485 UV/Vis detector at 254 nm equipped with SymmetryShieldTM RP18 column (5 μm, 4.6 × 150 mm, Waters corporation, Milford, MA, USA) using a gradient elution with acetonitrile/water containing 0.1% phosphoric acid as the mobile phase (v/v), at a flow rate of 1 mL/min. Peak areas were determined electronically with Waters Empower 2 chromatography data system.

*Conditions for HPLC analysis of cationic moieties containing 1,4-DHP amphiphiles* ***1-7****:*

Synthesized compounds were analyzed with HPLC on Waters Alliance 2695 system and Waters 2485 UV/Vis detector at 254 nm equipped with Alltima CN column (5 μm, 4.6 × 150 mm, Grace, Columbia, MD, USA) using gradient elution with acetonitrile in water containing 0.1% phosphoric acid as the mobile phase (v/v), at a flow rate of 1 mL/min. Peak areas were determined electronically with Waters Empower 2 chromatography data system.

*General methods*

All reagents were purchased from Acros Organics, Sigma-Aldrich, Alfa Aesar, or Merck KGaA and used without further purification. TLC was performed on silica gel 60 F254 aluminium sheets 20 × 20 cm (Merck KGaA). Silica gel of particle size 35–70 μm (Merck KGaA) was used for flash chromatography. Melting points were recorded on an OptiMelt digital melting point apparatus and are uncorrected. One-dimensional $^{1}H$ and $^{13}C$ NMR spectra were recorded at 400 MHz ($^{1}H$) and 100 MHz ($^{13}C$) operating frequencies with a Varian Mercury plus 400 or Varian 400-MR. Chemical shifts of the hydrogen and carbon atoms are presented in parts per million (ppm) and referred to the residual signals of the non-deuterated $CDCl_3$ (δ: 7.26) or partially deuterated DMSO-$d_6$ (δ: 2.50) solvent for $^{1}H$ NMR spectra and $CDCl_3$ (δ: 77.0) or DMSO-$d_6$ (δ: 39.5) solvent for $^{13}C$ NMR, respectively. Coupling constants, J were reported in hertz (Hz). Low resolution mass spectra (MS) were determined on an Acquity UPLC system (Waters) connected to a Waters SQ Detector-2 operating in the ESI positive or negative ion mode on a Waters Acquity UPLC® BEH C18 column (1.7 μm, 2.1 × 50 mm, using gradient elution with acetonitrile (0.01% trifluoroacetic acid) in water (0.01% trifluoroacetic acid). Elemental analyses were determined on an Elemental Combustion System ECS 4010 (Costech Instruments) at Laboratory of Chromatography of Latvian Institute of Organic Synthesis.

*Characterisation of original compounds.*

**1,1'-((3,5-Bis((dodecyloxy)carbonyl)-4-(naphthalen-2-yl)-1,4-dihydropyridine-2,6-diyl)bis(methylene))bis(pyridin-1-ium) dibromide (7)**

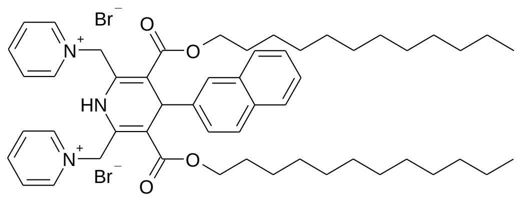

Yield: 55%; $T_{decomp.}$ 180-182°C, $^1$H NMR (400 MHz, $CDCl_3$, δ): 0.86 (t, 6H, J = 6.3 Hz, 3,5-$CH_3$); 1.10-1.29 (m, 36H, 3,5-$(CH_2)_9$); 1.55 (quint, 4H, J = 6.3 Hz, 3,5-$OCH_2\underline{CH_2}$); 4.00 (t, 4H, J = 6.3 Hz, 3,5-$OCH_2$); 5.22 (s, 1H, 4-H); 6.02 and 6.36 (AB-system, 4H, J = 13.7 Hz, 2,6-$CH_2$); 7.38-7.45 (m, 3H, 4-Ar); 7.65 (s, 1H, 4-Ar); 7.73-7.79 (m, 3H, 4-Ar); 8.20 (dt, 4H, J = 7.8 and 5.9 Hz, 3H-Py); 8.62 (t, 2H, J = 7.8 Hz, 4H-Py); 9.38 (d, 4H, J = 5.9 Hz, 2H-Py); 11.00 (br s, 1H, N-H);). $^{13}$C NMR ($CDCl_3$, δ): 14.04; 22.60; 25.97; 28.38; 29.24; 29.28; 29.29; 29.47; 29.59; 29.60; 31.84 (4-C-DHP); 40.00; 57.47 (2,6-$CH_2$-DHP); 65.41 (3,5-$OCH_2$); 109.88 (3,5-C-DHP); 125.94; 126.22; 126.75; 127.50; 127.79; 128.40; 128.73; 132.57; 133.19; 138.20; 142.95; 144.87; 144.95; 146.54; 166.32 (C=O). MS (+ESI) m/z (relative intensity) 816 ($^{79}$Br) ([M-2Br]+, 20%; 408 ([M-2Br]+/2, 100%). Anal. calcd for $C_{53}H_{73}NO_4Br_2$: C, 55.22; H, 7.54; N, 4.31; found: C, 55.60; H, 7.59; N, 4.16.

**Didodecyl 2,6-dimethyl-4-(naphthalen-2-yl)-1,4-dihydropyridine-3,5-dicarboxylate (7X)**

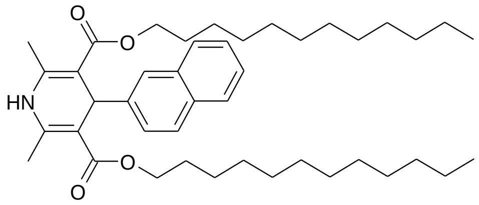

Yield: 39%; $T_{decomp.}$ 42°C, $^1$H NMR (400 MHz, $CDCl_3$, δ): 0.89 (t, 6H, J = 6.7 Hz, 3,5-$CH_3$); 1.19-1.32 (m, 36H, 3,5-$(CH_2)_9$); 1.58 (quint, 4H, J = 6.7 Hz, 3,5-$OCH_2\underline{CH_2}$); 2.37 (s, 6H, 2,6-$CH_3$); 4.00 and 4.03 (two t, 4H, J = 6.7 Hz, 3,5-$OCH_2$); 5.17 (s, 1H, 4-H); 5.68 (br s, 1H, N-H); 7.35-7.42 (m, 2H, 4-Ar); 7.47 (dd, 4H, J = 8.6 and 1.8 Hz, 4-Ar); 7.65-7.67 (m, 1H, 4-Ar); 7.69 (d, 2H, J = 8.6 Hz, 4-Ar); 7.72-7.76 (m, 2H, 4-Ar). $^{13}$C NMR ($CDCl_3$, δ): 14.27; 19.81; 22.85; 26.29; 28.91; 29.50; 29.52; 29.69; 29.78; 29.80; 29.82; 32.08; 39.97 (4-C-DHP); 64.14 (3,5-$OCH_2$); 104.27 (3,5-C-DHP); 125.21; 125.66; 126.31; 127.09; 127.58; 127.63; 127.97; 132.45; 133.48; 144.06; 145.22; 167.80 (C=O). MS (+ESI) m/z (relative intensity) 660 ([M]+, 100%). Anal. calcd for $C_{43}H_{65}NO_4$: C, 78.25; H, 9.93; N, 2.12; found: C, 77.87; H, 9.88; N, 2.00.

**1,1'-((3,5-Bis((dodecyloxy)carbonyl)-1-methyl-4-phenyl-1,4-dihydropyridine-2,6-diyl)bis(methylene))bis(pyridin-1-ium) dibromide (4)**

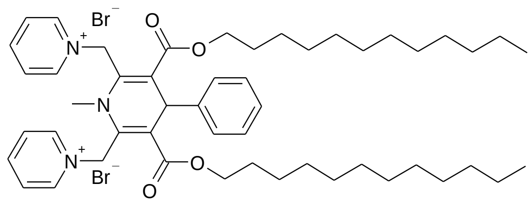

Yield: 50%; (38% [1]) $T_{decomp.}$ 179°C. $^{1}H$ NMR (400 MHz, DMSO-$d_6$, δ): 0.83 (t, 6H, J = 6.7 Hz, 3,5-$CH_3$); 1.18-1.26 (m, 36H, 3,5-$(CH_2)_9$); 1.52-1.59 (m, 4H, 3,5-$OCH_2\underline{CH_2}$); 3.11 (s, 3H, N-$CH_3$); 4.09 (t, 4H, J = 6.1 Hz, 3,5-$OCH_2$); 5.13 (s, 1H, 4-H); 5.80 and 6.47 (AB-system, 4H, J = 16.0 Hz, 2,6-$CH_2$); 7.20-7.31 (m, 5H, 4-Ar); 8.01 (dd, 4H, J = 7.8 and 5.9 Hz, 3-H Py); 8.49 (t, 2H, J = 7.8 Hz, 4-H Py); 8.85 (d, 4H, J = 5.9 Hz, 2-H Py). $^{13}C$ NMR (DMSO-$d_6$, δ): 13.73; 21.89; 25.43; 27.79; 28.53; 28.54; 28.82; 28.89; 28.94; 31.10 (N-$CH_3$); 33.65 (4-C-DHP); 55.27 (2,6-$CH_2$-DHP); 64.91 (3,5-$OCH_2$); 113.77 (3,5-C-DHP); 126.86; 126.93; 128.23; 128.42; 141.20; 142.53; 144.04; 146.12; 165.43 (C=O). Anal. calcd for $C_{50}H_{73}N_3O_4Br_2 \times 3H_2O$: C, 60.42; H, 8.01; N, 4.23; found: C, 60.48; H, 7.96; N, 4.08.

**Didodecyl 1,2,6-trimethyl-4-phenyl-1,4-dihydropyridine-3,5-dicarboxylate (4C)**

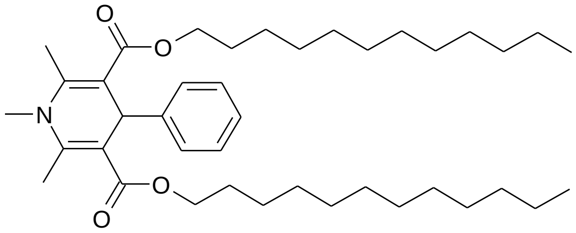

Yield: 63%; T 60-62°C, $^{1}H$ NMR (400 MHz, $CDCl_3$, δ): 0.88 (t, 6H, J = 6.7 Hz, 3,5-$CH_3$); 1.23-1.33 (m, 36H, 3,5-$(CH_2)_9$); 1.63 (quint, 4H, J = 6.7 Hz 3,5-$OCH_2\underline{CH_2}$); 2.48 (s, 6H, 2,6-$CH_3$); 3.18 (s, 3H, N-$CH_3$); 4.08 (t, 4H, J = 6.7 Hz, 3,5-$OCH_2$); 5.17 (s, 1H, 4-H); 7.09-7.21 (m, 5H, 4-Ar). $^{13}C$ NMR ($CDCl_3$, δ): 14.12; 16.36; 22.69; 26.11; 28.75; 29.32; 29.37; 29.60; 29.64; 29.69; 29.70; 29.72; 31.93 (4-C-DHP); 38.21; 64.07 (3,5-$OCH_2$); 106.30 (3,5-C-DHP); 126.02; 126.98; 127.00; 146.14; 149.29; 168.00 (C=O). Anal. calcd for $C_{40}H_{67}NO_4$: C, 77.00; H, 10.50; N 2.24; found: C, 77.01; H, 10.53; N, 2.24.

*Results of thermal analysis of 1,4-DHP amphiphiles*

**Table S1.** Temperatures characteristics of tested compounds **1–7,** obtained by analysing TGA and DTA curves. The compounds were heated from 30 to 300°C; heating rate 5°C/min, symbol "↓" denotes endothermic transition.

| Comps. | Thermogravimetric Analysis | | Differential Thermal Analysis | | | |
|---|---|---|---|---|---|---|
| | Temp. range, °C | Weight loss, % | Transition | Temp. Range, °C | Peak Temp., °C | Absorbed Heat, J/g |
| **1** | 30–98.5 | −0.243 | 1nd ↓ | 53.5–60.2 | 55.8 | −20.2 |
| | | | 2rd ↓ | 73.4–84.4 | 79.2 | −94.3 |
| | | | 3rd ↓ | 86.6–92.9 | 88.6 | −77.8 |
| | 30–212 | −55.7 | 4th ↓ | 153.3–198.5 | 157.8 | $-2.43{\cdot}10^3$ |
| **2** | 30–74.8 | −1.162 | 1st ↓ | 41.4–48.8 | 43.8 | −10.05 |
| | | | 2nd ↓ | 55.4–74.5 | 59.8 | −173.9 |
| | 30–200 | −57.52 | 3rd ↓ | 153–200 | 187.7 | $-1.91{\cdot}10^3$ |
| **3** | 30–80.3 | −2.12 | 1st ↓ | 47–56.7 | 53.7 | −80.35 |
| | | | 2nd ↓ | 59.8–67.9 | 63.8 | −45.3 |
| | 30–219 | −60.8 | 3th ↓ | 150.2–198.5 | 157.8 | $-2.19{\cdot}10^3$ |
| **4** | 30–60 | −1.66 | 1st ↓ | 43.4–59.5 | 54.2 | −144.47 |
| | | | 2nd ↓ | 61.8–79.1 | 72.9 | −171.34 |
| | 30–209 | −39.1 | 3rd ↓ | 194.8–208.6 | 200 | $-1.65{\cdot}10^3$ |
| **5** | 30–177 | −68.2 | 1st ↓ | 152.1–176.7 | 159.5 | $-2.43{\cdot}10^3$ |
| **6** | 30–73 | −1.85 | 1st ↓ | 67.0–72.8 | 69.2 | −447.98 |
| | 30–201 | −48. 51 | 2nd ↓ | 180.3–201.2 | 189.95 | $-2.20{\cdot}10^3$ |
| **7** | 30–67 | −0.52 | 1st ↓ | 60.9–67.0 | 63 | −51.75 |
| | 30–202 | −40.65 | 2nd ↓ | 165.5–202 | 177 | $-1.64{\cdot}10^3$ |

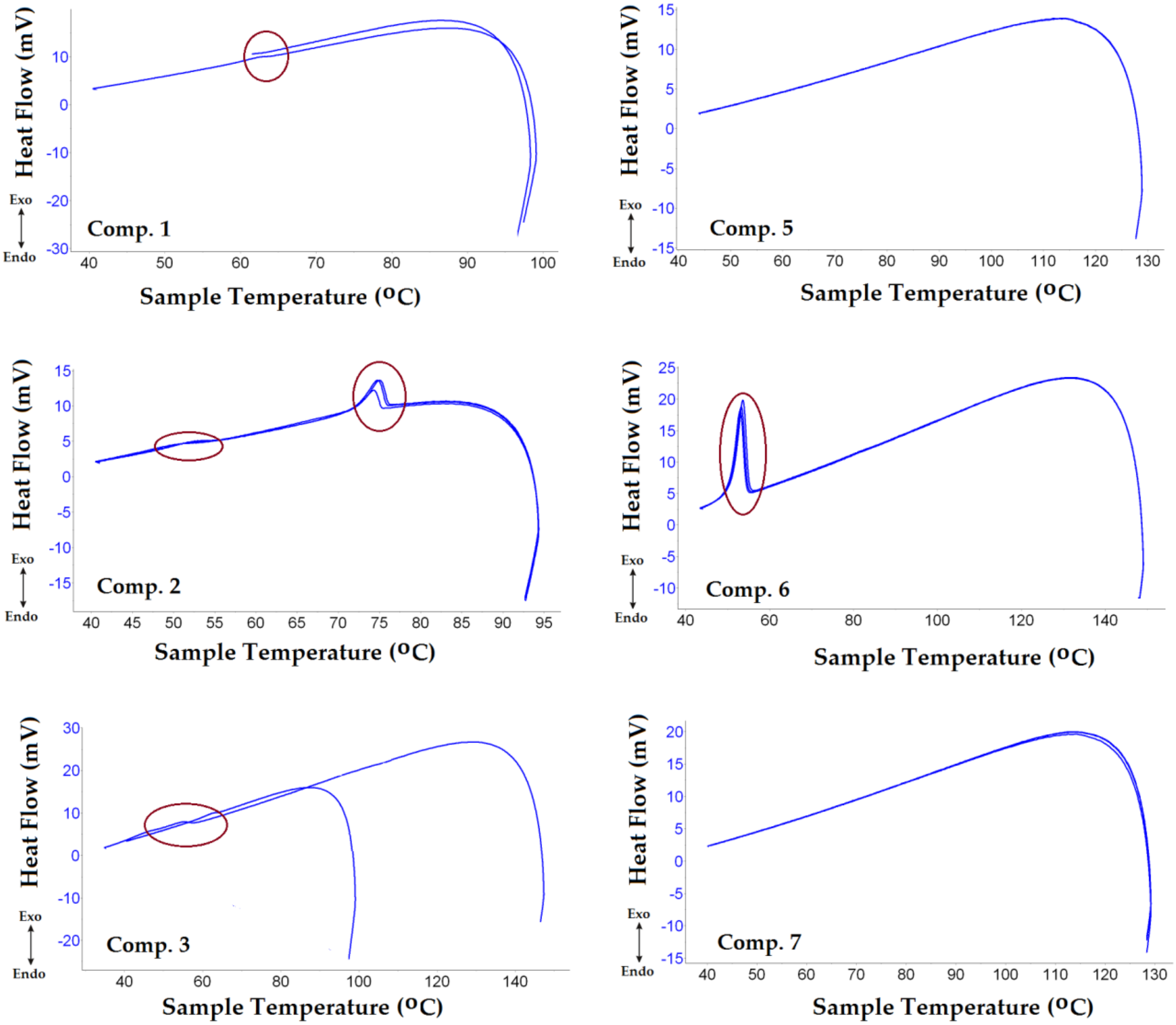


**Figure S1.** Cooling curves obtained by DSC after heating process for tested compounds **1**–3, **5**–**7**.

*Surface pressure-area isotherms, mechanical properties of monolayers*

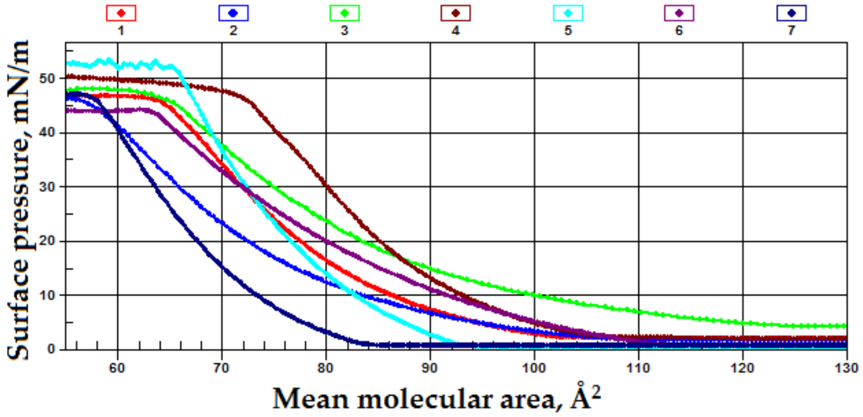


**Figure S2.** 1,4-DHP amphiphiles **1–7** surface pressure – mean molecular area isotherms at 23±1°C.

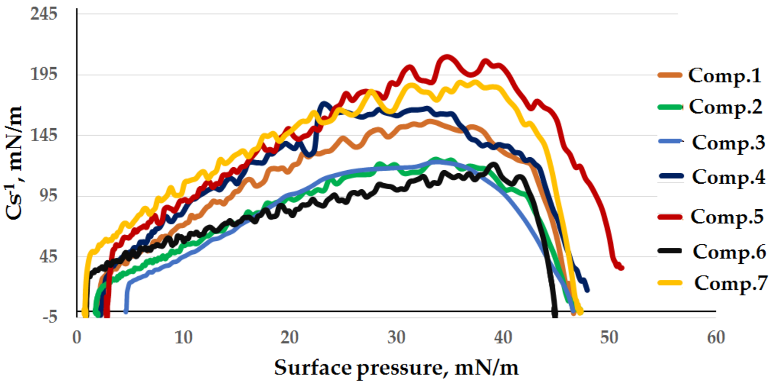


**Figure S3.** Compressibility modulus-surface pressure dependences obtained for the 1,4-DHP amphiphiles **1–7** monolayers.

*Characterization of liposomes by dynamic light scattering*

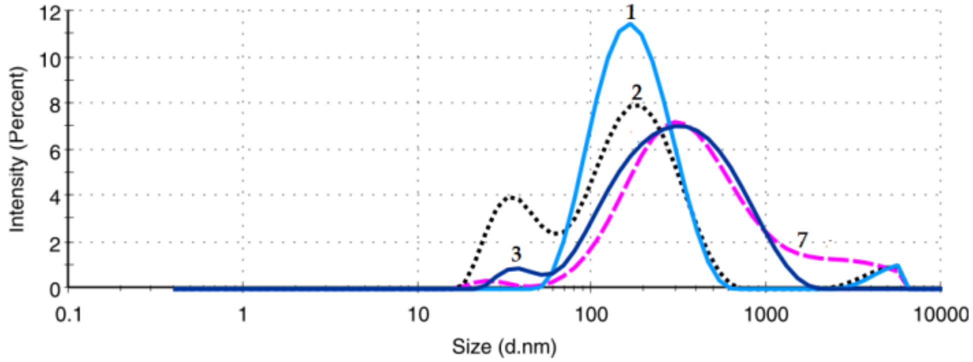


**Figure S4.** Hydrodynamic size distribution of the 'empty' liposomes formed by 1,4-DHP amphiphiles **1–3** and **7**. Liposomes obtained by REV.

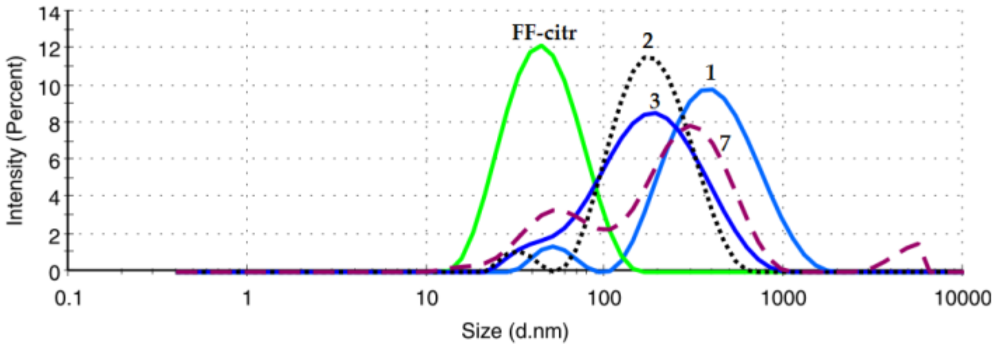


**Figure S5.** Hydrodynamic size distribution of the magnetoliposomes formed by 1,4-DHP amphiphiles **1–3** and **7**. Liposomes obtained by REV: 1,4-DHP amphiphile and FF-citr). Curve FF-citr demonstrate the NPs hydrodynamic size distribution in the FF-citr used for liposomes preparation.

## References


[1] Hyvönen, Z.; Plotniece, A.; Reine, I.; Chekavichus, B.; Duburs, G.; Urtti, A. Novel cationic amphiphilic 1,4-dihydropyridine derivatives for DNA delivery. *Biochim. Biophys. Acta*. **2000** *1509*, 451–466. doi:10.1016/S0005-2736(00)00327-8

[2] Pajuste, K.; Hyvönen, Z.; Petrichenko, O.; Kaldre, D.; Rucins, M.; Cekavicus, B.; Ose, V.; Skrivele, B.; Gosteva, M.; Morin-Picardat, E.; Plotniece, M.; Sobolev, A.; Duburs, G.; Ruponen, M.; Plotniece, A. Gene delivery agents possessing antiradical activity: self-assembling cationic amphiphilic 1,4-dihydropyridine derivatives. *New J. Chem*. **2013** *37*, 3062–3075. DOI:10.1039/C3NJ00272A.

[3] Pajuste, K.; Plotniece, A.; Kore, K.; Intenberga, L.; Cekavicus, B.; Kaldre, D.; Duburs, G.; Sobolev, A. Use of pyridinium ionic liquids as catalysts for the synthesis of 3,5-bis(dodecyloxycarbonyl)-1,4-dihydropyridine derivative. *CEJC*. **2011** *9*, 143–148. DOI: 10.2478/s11532-010-0132-x
